# Volatile Compound-Mediated Interactions between Barley and Pathogenic Fungi in the Soil

**DOI:** 10.1371/journal.pone.0066805

**Published:** 2013-06-20

**Authors:** Marie Fiers, Georges Lognay, Marie-Laure Fauconnier, M. Haïssam Jijakli

**Affiliations:** 1 Phytopathology Unit, Gembloux Agro-Bio Tech (GxABT), University of Liège, Gembloux, Belgium; 2 Analytical Chemistry Laboratory, Gembloux Agro-Bio Tech (GxABT), University of Liège, Gembloux, Belgium; 3 General and Organic Chemistry Unit, Gembloux Agro-Bio Tech (GxABT), University of Liège, Gembloux, Belgium; Virginia Tech, United States of America

## Abstract

Plants are able to interact with their environment by emitting volatile organic compounds. We investigated the volatile interactions that take place below ground between barley roots and two pathogenic fungi, *Cochliobolus sativus* and *Fusarium culmorum*. The volatile molecules emitted by each fungus, by non-infected barley roots and by barley roots infected with one of the fungi or the two of them were extracted by head-space solid phase micro extraction and analyzed by gas chromatography mass spectrometry. The effect of fungal volatiles on barley growth and the effect of barley root volatiles on fungal growth were assessed by cultivating both organisms in a shared atmosphere without any physical contact. The results show that volatile organic compounds, especially terpenes, are newly emitted during the interaction between fungi and barley roots. The volatile molecules released by non-infected barley roots did not significantly affect fungal growth, whereas the volatile molecules released by pathogenic fungi decreased the length of barley roots by 19 to 21.5% and the surface of aerial parts by 15%. The spectrum of the volatiles released by infected barley roots had no significant effect on *F. culmorum* growth, but decreased *C. sativus* growth by 13 to 17%. This paper identifies the volatile organic compounds emitted by two pathogenic fungi and shows that pathogenic fungi can modify volatile emission by infected plants. Our results open promising perspectives concerning the biological control of edaphic diseases.

## Introduction

Smell emission and detection, though relatively unknown, is a major means for plants to interact with other organisms within their environment. Since the 1980’s, the scientific community has been interested in volatile organic compounds (VOCs), which are secondary metabolites produced by plants, and in their effects on their biological environment [1]. Thanks to their low molecular weight, their lipophilic nature and their high vapour pressure, some VOCs can freely cross membranes and be released into the atmosphere. In a way, they act as a kind of language for plants. Plants are known to emit airborne VOCs to attract pollinators and seed dispersers and to prevent herbivores’ and pathogens’ attacks [2], [3]. Some VOCs induce direct or indirect defences through tritrophic plant-herbivore-carnivore interactions. Plants under herbivore attack emit VOCs that attract specific enemies of the same herbivores. For example, corn seedlings attacked by caterpillars release large amounts of terpenoid volatiles that repel herbivores and allow parasitic wasps to locate potential hosts [4], [5]. Unaffected plants in the vicinity are also able to recognize volatiles emitted by infected or attacked plants and react by emitting defence volatiles [6]. Very precise signals can be emitted by plants according to the kind of noxious organism they meet. But the particular response also depends on the ability of the organisms to discriminate between different odour profiles [7]. The study of below-ground emissions of VOCs started only recently. However, volatiles emitted from roots are already known to contribute to the defence system by acting as antimicrobial and anti-herbivore substances, or by attracting enemies of root-feeding herbivores [8]. However scientific literature is rather poor concerning fungal infections. In this paper, the below-ground volatile interactions between plant roots and pathogenic fungi are investigated. The chosen models are barley and two pathogenic fungi, *Cochliobolus sativus* and *Fusarium culmorum*.

Barley has high agronomical significance (123 million tons produced in the world in 2010) and is sensitive to many diseases (40 pathogens inducing an average loss of $ 252 million *per* year, *i.e.* 19.6% of the average annual value of barley crop) [9], [10]. *Cochliobolus sativus* (Ito and Kuribayashi) Drechsler ex. Dastur, 1942 [anamorph *Bipolaris sorokiniana* (Sacc.), Shoemaker] [11] and different *Fusarium* species, especially *F. culmorum* (W.G. Smith, 1892, Sacc.) are the major seed-borne and soil-borne pathogenic causal agents of common root rot of barley. This major edaphic disease of cereals causes between 9 and 23% yield losses, depending on growth area and cultivars [12]–[14]. Primary symptoms are seedling blight sometimes leading to sprout death. The advanced stages of the diseases show dark-brown lesions on the outer coleoptile tissue and/or on the leaves and head blight, *i.e.* premature head death or blighting spikelets [15], [16]. The VOCs emitted by fungi can contribute to the success of an infection since they are lethal for some other fungi and act as pathogenic factors by inhibiting plant growth or being toxic for plants [17], [18].

The objective of this paper is to show what kind of volatile interactions can take place between barley and the pathogenic fungi *C. sativus* and *F. culmorum*. Firstly, the VOCs produced by each organism and during the interaction between the two organisms were identified. Secondly, the effects of the total VOC emission from barley root on fungi, and *vice versa* were assessed.

## Materials and Methods

### 1. Fungal Strains

*F. culmorum* (MUCL 28166) and *C. sativus* (MUCL 46854) strains were provided by the Belgian Co-ordinated Collection of Microorganisms (BCCM – MUCL) (Louvain-la-Neuve, Belgium)**.** They were stored on PDA medium (Merck KGaA, Darmstadt, Germany) at 23°C and by cryopreservation at −80°C.

### 2. Plant Material

Barley plants (*Hordeum vulgare* L. *cv.* ‘Quench’) were grown *in vitro* from seeds (Jorion, Belgium). Before use, they were surface-sterilized according to a protocol adapted from Lanoue *et al.* [19] with incubation in H_2_SO_4_ (10% v/v). They were placed on Hoagland agar medium (1.6 g/L Hoagland, Plantmedia, Dublin, Ohio, USA; 1% agar, BD-Difco, Franklin Lakes, New Jersey, USA) for 48 hours at 23°C for pregermination until roots emerged.

### 3. Volatile Organic Compounds Analyses

#### 3.1. Sample preparation for VOCs analyses

In order to analyse the blend of VOCs emitted by the two fungi and by non-infected or infected barley roots, samples of each organism were prepared. Fungi were grown on PDA medium slants. Barley roots were grown *in vitro* on Hoagland agar medium for 7 days.

Conidial suspensions of *F. culmorum* or *C. sativus* were prepared by pouring 1 or 4 ml of sterile water on PDA plates colonized by *C. sativus* or *F. culmorum*, respectively. The mycelium was gently scratched with a sterile scalpel and the water-and-conidia mix was filtered on a double layer of cheesecloth placed on a sterile funnel. This operation was repeated as many times as necessary to obtain the required volume of filtrate. The concentrations of the suspensions were determined by counting conidia on a Fuchs-Rosenthal counting chamber (Hecht Assistent, Sondheim/Rhön, Germany) according the manufacturer’s instructions. The concentrations were adjusted at 2×10^3^ conidia/mL for fungal VOC analyses and at 1×10^6^ conidia/mL for infected barley root analyses, using sterile water. In order to distribute the conidia evenly, 0.1% Tween 20 (Merck KGaA, Darmstadt, Germany) was added to each conidial suspension.

Fungal cultures were conducted on 7 mL PDA slants in 20 mL SPME vials (Filter Service, Eupen, Belgium) fitted with sealed caps (White silicone/blue PTFE, Filter Service). A drop of 50 µL of conidial suspension at 2×10^3^ conidia/mL was poured onto the slant and spread across the whole surface (about 40 cm^2^) with a loop. The caps on the vials were not fully closed to allow for oxygen supply. The vials were placed in a growth chamber, at 23°C +/−2°C. Five replicates of each fungal culture were prepared. The 5 replicates of *C. sativus* were prepared and analysed on the same day, but the replicates 1, 2, and 3 and the replicates 4 and 5 of *F. culmorum* were analysed at two different periods of time after an interval of 50 days. Empty vials and vials containing only PDA were used as controls. VOCs analyses were carried out 2, 7, 14, 21 and 27 days after inoculation (dai) for *F. culmorum* and 2, 7, 14, 21 and 28 dai for *C. sativus*.

Non-infected and infected barley roots were obtained by growing barley on square Petri dishes (Greiner, Belgium, 12 cm) containing 50 mL of Hoagland agar medium (1% v/v). After 2 days germination, 10 sterile barley seeds *per* Petri dish were placed in a row, 3 cm from the edge. For the production of infected roots, 10 seeds were infected by spraying 1 mL of *F. culmorum* and/or *C. sativus* suspension (1×10^6^ conidia/mL) onto the Petri dishes with a spray gun (Badger 350, Badger, Illinois, USA). A control treatment was carried out by spraying seeds with 0.1% Tween 20 in 1 mL of sterile water. Dishes of non-infected or infected roots were hermetically closed and placed vertically, plant roots pointing downward, in a growth chamber under LED light (94 µmol photons/m^2^/s) with a 20 h/4 h photoperiod at 22°C +/−0.5°C for 7 days. Five replicates of each treatment were produced and analysed strictly in the same conditions, in two independent experiments after an interval of 44 days, except for roots infected by the two fungi for which the 5 replicates were produced and analysed on the same day. After 7 days, the plants were gently pulled off the Hoagland agar medium and the roots were excised with scissors just under the seed. The roots of 10 plants were placed into a 20 mL SPME vial. Empty vials and vials with a piece of Hoagland agar medium were used as controls. After analyses, barley roots were dried in a drying oven at 150°C during 24 h and weight.

#### 3.2. Head-Space Solid-Phase-Micro-Extraction (HS-SPME)

The Solid-Phase-Micro-Extraction (SPME) technique consists in catching the VOCs contained in the head-space above a sample in an SPME vial with a fibre coated with adapted stationary phases.

SPME fibres were coated with three 65 µm thick stationary phases : divinylbenzene – carboxen - polydimethylsiloxane (DVB – Carboxen - PDMS) (Supelco, Bellefonte, PA, USA). All fibres were conditioned before first use at 270°C for 1 hr according to the supplier’s recommendations and they were tested before each analysis to minimize the variations due to the fibre deterioration. They were tied to a plunger within a protective needle that directly pierced through vial septa and were exposed to the head-space within the sample vials. Internal standards (1µL of a methanolic solution of butylbenzene (≥99%, Sigma-Aldrich, Belgium) at 86.6 ng/µL for fungal analyses and at 8.66 ng/µL for root analyses were added into each vial using a 1-µL-gas-tight calibrated Hamilton syringe. Briefly, SPME extractions were performed using the following method : after equilibration of the vials for 10 min at 25°C, the fibre was inserted into the head-spaces for 20 min at the same temperature. After extraction, the volatile compounds were desorbed in pulsed splitless mode for 5 min at 250°C.

#### 3.3. GC-MS analyses

Gas chromatography–mass spectrometry (GC-MS) analyses were performed on a gas chromatograph (Agilent Technologies 7890A) coupled to a mass selective detector (Agilent Technologies 5975 C) with enhanced Chemstation version E.02.00.493 software. The GC, fitted with a Gerstel MPS autosampler (Maestro 1, version 1.4.8.14/3.5; Müllheim/Ruhr, Germany), was used for SPME injection and it was checked weekly for its resolution capacity and the conservation of retention times. Separation was performed on a VF-WAX column (Agilent technologies, USA; 30 m x 0.250 mm I.D, 0.25 µm film thickness). Helium was used as a carrier gas at a constant flow rate of 1.5 mL/min. The inlet temperature was 250°C. Pulsed splitless injection mode in a 1.5 mm HS-liner was used (injection pulse pressure of 30 psi for 1 min). The following temperature programs were used: 35°C for 2 min, 5°C/min up to 155°C, 20°C/min up to 250°C, and a final hold at 250°C for 10 min. The mass spectrometer was operated in electron ionization (EI) mode at 70 eV, source temperature 230°C; quadrupole temperature 150°C; scanned mass range from 20 to 350 amu, threshold of 150 amu; scan speed, 4.27 scans/sec. It was regularly calibrated in order to produce ions of exact mass and constant response intensity in time.

#### 3.4. Compounds identification

VOC identification was undertaken by comparing retention times and recorded mass spectra using the Wiley 275, pal600k and NBS75K spectral databases. Further identification was carried out by comparing the theoretical non-isothermal Kovats retention indices of each molecule with calculated retention indices. Retention indices were calculated by injecting saturated n-alkane standard solution C_7_–C_30_ (1,000 µg/mL in hexane, Supelco, Belgium), using the definition of Van den Dool and Kratz [20].

Whenever possible, identifications were confirmed by injecting available commercial standards provided by Sigma-Aldrich with chromatographic purity, except n-hexane (Scharlau; ≥96%); heptane (VEL; ≥99%); octa-1,3-diene (Interchim); p-xylene and m-xylene (UCB chemicals); butan-1-ol (Janssen Chimica; 99.5%); longifolene (Penta Manufacturing); (2E,6Z)-nona-2,6-dienal (Interchim) and tetradecanal (TCI; >95%). A mix was made from pure concentrated standards by diluting standards 105 fold in methanol (concentration range from 65.9 to 101.7 mg/L). For GC-MS measurements, 1µL of a standard mix was placed on the walls of a 20 mL vial before quickly closing it securely. Only the 5 major non-identified VOCs with a relative quantity higher than the limit of quantification (i.e. signal/noise ratio >3 at the same retention time) were included in the results.

#### 3.5. Quantification

GC-MS analyses only allow for relative quantifications of emitted VOCs. Relative quantities were calculated by comparing the area of the butylbenzene peak, whose quantity was known (86.6 or 8.66 ng), with the area of the peak of interest. They were expressed as mean percentages of total relative VOC quantities. For barley root analyses, the mean percentages were standardized for 1 gram of dry roots.

### 4. *In vitro* Co-culture of Fungi and Barley

**Figure 1:**
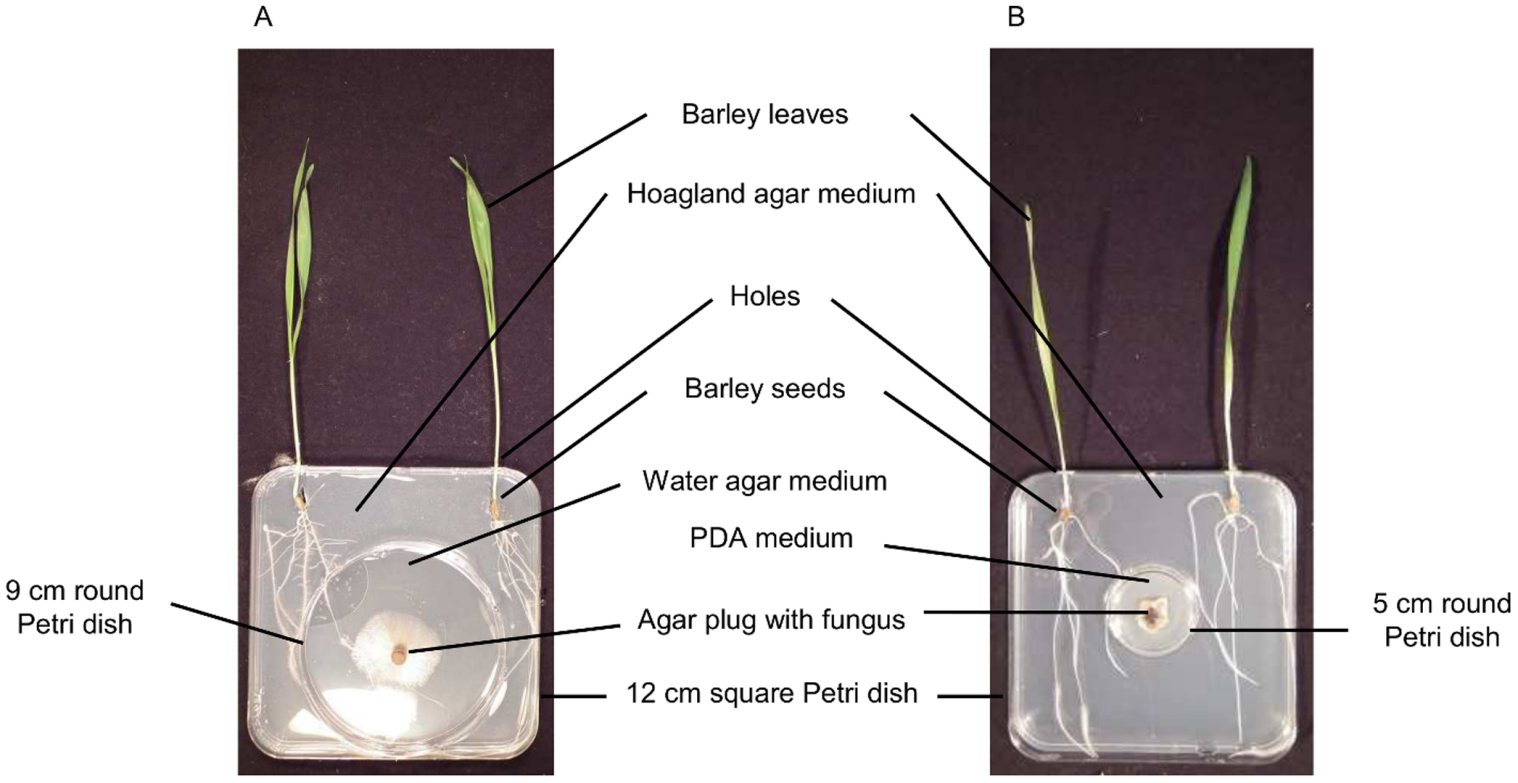
Pictures of the barley and fungi co-culture devices. Experimental devices for *in vitro* co-culture for the study of the effects of VOCs from non-infected or infected barley roots on pathogenic fungi (A) and for the study of the effects of fungal VOCs on barley (B).

In order to investigate the effect of fungal VOCs on barley and *vice versa*, barley and fungi were grown in different compartments of a co-culture device, sharing the same atmosphere. VOCs were thus the only way the two organisms could communicate. Moreover, barley leaves were removed from the co-culture device in order to only take into account the VOCs emitted by the roots (Figure 1).

#### 4.1. Effects of the blend of VOCs from non-infected barley roots on pathogenic fungi

To assess the effects of the spectrum of VOCs emitted by barley roots on fungi, the co-culture device consisted of 9-cm round Petri dishes without a lid (VWR, Belgium) placed in 12-cm square Petri dishes (Greiner, Belgium). Square Petri dishes were higher than round Petri dishes to allow VOCs to diffuse freely within the entire experimental device. Twenty-five mL of sterile water agar (1% w/v) were poured into each round Petri dish and 45 mL of Hoagland agar medium (1% w/v) were poured into each square Petri dish around the round Petri dish. Two holes were made with a heated puncher through each square Petri dish and lid to allow barley leaves to develop outside. The holes were filled with sterile cotton wool until the leaves were long enough to be taken out of the Petri dish (Figure 1A).

Two surface-sterilized barley seeds germinated for 48 hrs were placed in front of each hole. A 7-mm round plug of agar colonized by a 5-days old *F. culmorum* or a 14-days old *C. sativus* was placed in the center of each round Petri dish. Four treatments were tested: *F. culmorum* with or without barley and *C. sativus* with or without barley. Three independent trials were set up, with 10 replicates *per* treatment. The co-culture devices were sealed with parafilm and placed on a rack in a quasi-vertical position in a climatic chamber at 23°C. After 24 or 48 hrs, when barley leaves reached 2 cm, they were taken out of the co-culture device through the previously bored holes. Holes were filled as much as possible around the coleoptile using parafilm.

The diameter of each fungus was measured 24, 48, 72, 96, 168 and 192 hours after inoculation (hai).

#### 4.2. Effects of the blend of VOCs from infected barley roots on pathogenic fungi

The experimental device was the same as previously described. Each barley seed was inoculated with a conidial suspension of *F. culmorum* and *C. sativus*. Conidial suspensions were prepared as described in part 2.4.1. The concentration of each conidial suspension was adjusted at 1⋅10^6^ conidia/mL with sterile water and 0.1% Tween 20 was added. About 100 µL of conidial suspension were sprayed on each barley seed as previously described. For each fungus, roots were infected with *F. culmorum* (5 replicates), *C. sativus* (5 replicates) or water with 0.1% Tween 20 as a control (5 replicates).

#### 4.3. Effects of the blend of VOCs from pathogenic fungi on barley

The co-culture device was almost the same as described in part 2.4.1. It consisted of 5-cm round Petri dishes without a lid (VWR, Belgium) containing 10 mL of PDA, placed at the center of 12-cm square Petri dishes (Greiner, Belgium) containing 50 mL of Hoagland 1% agar medium (Figure 1B).

Three different treatments were tested: barley cultivated alone or in the presence of *F. culmorum* or *C. sativus*. Four independent trials with 10 replicates *per* treatment were set up.

Two surface-sterilized barley seeds germinated 48 hrs earlier were placed in each square Petri dish, in front of each hole. A 7-mm diameter piece of agar colonized by a 5-days old *F. culmorum* was placed in the center of each round Petri dish. The same was done with a 14-days old *C. sativus*. Co-culture devices without fungi were used as controls. Co-culture devices were sealed with parafilm and placed on a rack in a quasi-vertical position in a climatic chamber at 23°C for 7 days. Barley leaves higher than 2 cm were taken out of the Petri dishes through the holes.

After 7 days, a picture and a scan of each co-culture device were taken. Plant aerial parts and roots were counted and weighed, and leaf surfaces and root lengths were measured.

### 5. Data Analysis

In each experiment, only the VOCs detected at the two dates of analysis were taken into account in order to minimize the variations due to the different dates of analysis. Thus the variations of VOC emission in time were minimized.

The data were analyzed using analysis of variance (*P*<0.05) performed under Excel software (Microsoft Office Excel, 2007), and Dunnett’s multiple comparisons test (α <0.05) were performed under Minitab 16.2.2 software [21]. Leaf surfaces were measured with MVHimage software (PC v.8, John Pickle and Jacqueline Kirtley, Museum of Science, Boston, MA, 2004).

## Results

### 1. Analysis and Identification of Fungal and Barley VOCs

#### 1.1. VOCs emitted by pathogenic fungi

**Figure 2:**
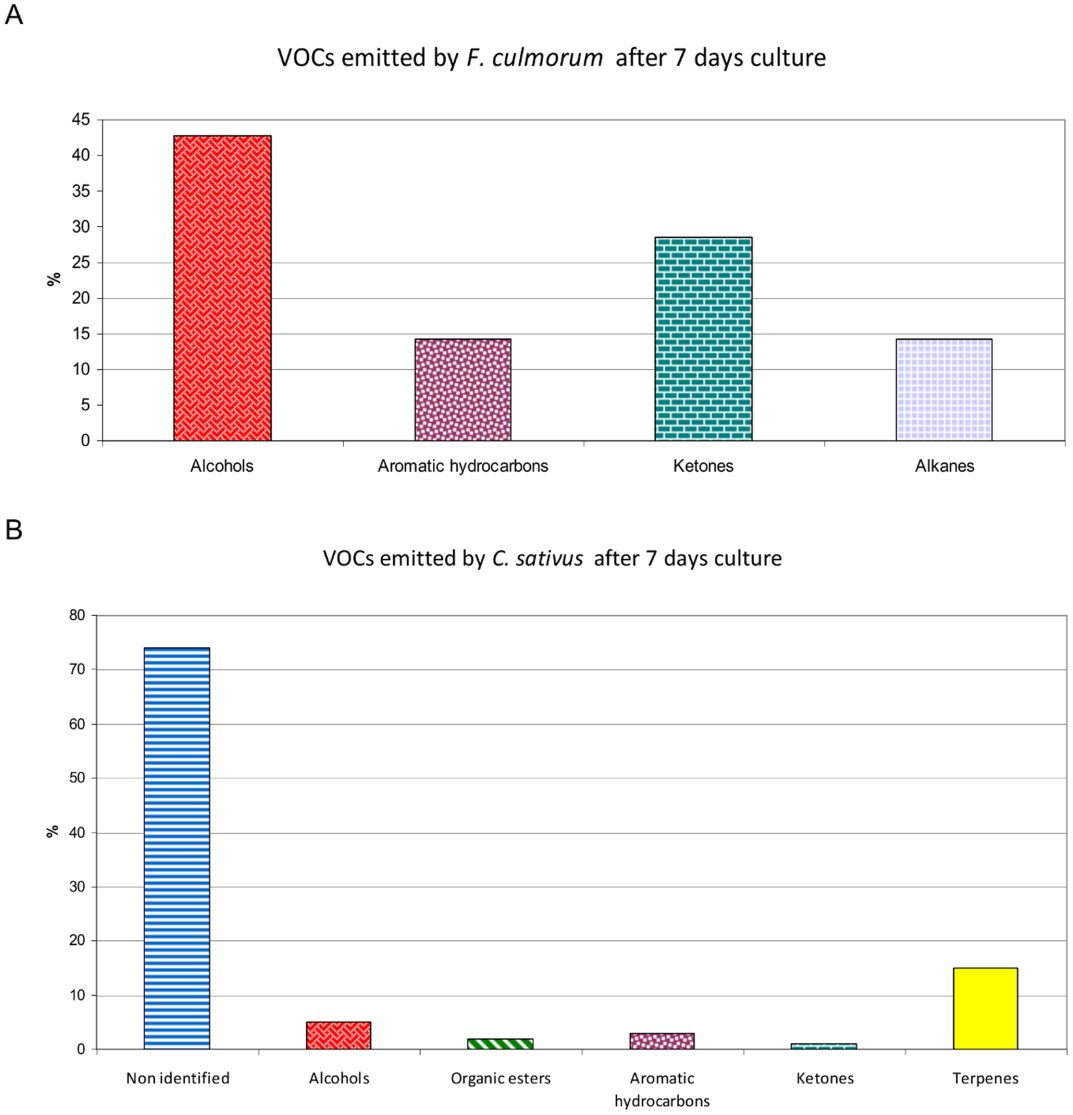
Chemical families of the VOCs emitted by *Fusarium culmorum* (A) and *Cochliobolus sativus* (B).

Biogenous fungal VOCs were determined by comparing the analyses of fungal VOCs with control analyses (column, fibre, empty vial and the vial containing agar medium). In order to compare fungal analysis with barley roots analysis, only the fungal VOCs released after 7 days *in vitro* culture were reported in Table 1. The VOCs emitted over time after 2, 7, 14, 21, and 27 or 28 days are reported in the table S1 data. The ANOVAs (P<0.05) comparing the variances of the 5 replicates of each experiment showed that there were significant differences between the replicates that were analysed at different periods of time, only after 2 days culture. The replicates 1, 2 and 3 were different from the replicates 4 and 5 for *C. sativus* (p<0.0001) and *F. culmorum* (p  = 0.005). At 7, 14, 21 and 27 or 28 days culture, the differences between the 5 replicates were not significant either for *C. sativus* (0.451<p<0.977) or for *F. culmorum* (0.079<p<0.351). Gas chromatography analyses showed that seven biogenous VOCs were emitted by *F. culmorum* after 7 days culture (Table 1 and Figure 2 A), and 13 were emitted after 27 days (Table S1). Alcohols represented 43% of the volatile diversity emitted by *F. culmorum* and 53% of the total relative quantity emitted in 7 days. Concerning *C. sativus*, 105 compounds were detected after 7 days culture and 141 after 28 days culture, most of them were not identified (Table 1, Figure 2 B and Table S1). Terpenes represented 15% of VOC diversity and 97% of the relative quantity emitted in 7 days. Non identified molecules represented 5% of the relative emitted quantity and at least 16 of them were terpenoid compounds.

**Table 1:** Percentages of VOCs emitted by the pathogenic fungi *Fusarium culmorum* and *Cochliobolus sativus*, by non-infected barley roots and by barley roots infected by *F. culmorum*, by *C. sativus* or by both.

						Emitted by					
Compounds^a^	CAS	RIcal^b^	RIref^c^	Identification^d^	Class^e^	*C. sativus* f	*F. culmorum* f	Non-infected roots g	*C. sativus*-infected roots g	*F. culmorum*-infected roots g	*C. sativus*+*F. culmorum* infected roots g
Pentane	109-66-0	500	500	MS, RI	Aa	–	–	0.97±0.60	0.11±0.05	0.16±0.12	–
n-hexane	110-54-3	600	600	MS, RI, STD	Aa	-	30.4±22.63	–	–	–	–
2-methylbuta-1,3-diene	78-79-5	665	-	MS	Ae	0.01±0.00	–	–	–	–	–
Methyl methanoate	107-31-3	736	682 [46]	MS, RI	E	–	–	–	3.36±3.37	-	17.65±2.25
Dimethyl sulphide	75-18-3	737	844 [47]	MS, RI, STD	S	–	–	1.2±0.26	0.75±0.43	0.35±0.26	0.15±0.06
Methyl acetate	79-20-9	814	864 [47]	MS, RI, STD	E	–	–	2.73±1.43	0.99±0.55	1.4±1.68	2.2±0.44
Ethyl acetate	141-78-6	826	898 [48]	MS, RI, STD	E	–	–	2.14±1.11	–	–	–
Methyl propanoate	554-12-1	889	911 [49]	MS, RI, STD	E	–	–	–	3.43±4.13	-	2.19±0.65
3-methylbutan-2-one	563-80-4	918	–	MS, STD	K	–	0.69±0.46	–	–	–	–
Methyl acrylate	96-33-3	938	–	MS, STD	E	–	–	–	1.75±0.97	–	1.06±0.09
Formic acid, 1-methylpropyl ester	**589-40-2**	941	–	MS	E	–	–	–	< LOQ	–	0.24±0.04
Octa-1,3-diene	1002-33-1	949	–	MS, STD	Ae	–	–	–	1.06±0.51	0.27±0.02	0.45±0.08
2-methylpropyl formate	542-55-2	953	–	MS, STD	E	0.01±0.003	–	–	2.32±0.6	–	3.75±0.33
Pentan-3-one	96-22-0	957	984 [50]	MS, RI, STD	K	–	–	3.4±0.23	0.73±0.15	1.31±0.39	1.08±0.30
Methyl butanoate	623-42-7	962	982 [51]	MS, RI, STD	E	–	–	0.55±0.43	0.36±0.16	–	0.31±0.05
Butanoic acid, 2-methyl, methyl ester	868-57-5	985	-	MS	E	–	–	0.83±0.29	-	0.68±0.47	0.41±0.09
Methyl 3-methylbutanoate	556-24-1	995	1015 [52]	MS, RI	E	–	–	1.08±0.65	0.78±0.13	0.43±0.37	0.61±0.21
2-methylbut-3-en-2-ol	115-18-4	1020	1036 [53]	MS, RI, STD	A	< LOQ	–	–	–	–	–
Compounds^a^	CAS	RIcal^b^	RIref^c^	Identification^d^	Class^e^	*C. sativus* f	*F. culmorum* f	Non-infected roots g	*C. sativus*-infected roots g	*F. culmorum*-infected roots g	*C. sativus*+*F. culmorum* infected roots g
NI #1^h^	–	1032	–	–	Aa	–	–	–	0.18±0.04	0.24±0.29	< LOQ
NI #2^h^	–	1041	–	–	Aa	–	–	–	0.18±0.02	< LOQ	-
Methyl pentanoate	624-24-8	1066	1087 [49]	MS, RI, STD	E	–	–	–	< LOQ	–	< LOQ
Undecane	1120-21-4	1100	1100	MS, RI	Aa	–	–	0.48±0.13	0.19±0.07	–	–
1,3-cis,5-cis-octatriene	33580-05-1	1102	1108 [54]	MS, RI	Ae	–	–	-	< LOQ	–	0.87±0.87
2-methylpropan-1-ol	78-83-1	1103	1108 [55]	MS, RI	A	< LOQ	27.2±11.07	-	< LOQ	–	0.3±0.14
Heptan-4-one	123-19-3	1108	-	MS, RI	K	–	-	-	< LOQ	1±0.59	0.69±0.09
para-xylene	106-42-3	1109	1127 [49]	MS, RI, STD	H	–	8.62±2.83	1.88±0.46	0.22±0.16	0.54±0.8	-
meta-xylene	108-38-3	1115	1132 [49]	MS, RI, STD	H	–	–	1.27±0.24	0.09±0.00	0.09±0.02	-
Butan-1-ol	71-36-3	1121	1145 [56]	MS, RI, STD	A	–	–	0.24±0.09	< LOQ	-	-
Pent-1-en-3-ol	616-25-1	1137	1176 [49]	MS, RI, STD	A	–	–	–	0.34±0.11	-	<LOQ
alpha-phellandrene	99-83-2	1137	1205 [57]	MS, RI	T	–	–	–	–	1.1±0.90	< LOQ
alpha-terpinene	99-86-5	1154	1208 [58]	MS, RI, STD	T	–	–	–	–	0.25±0.04	< LOQ
(E)-hex-2-enal	**6728-26-3**	1160	1220 [59]	MS, RI, STD	O	–	–	–	–	–	0.12±0.06
Methyl hexanoate	106-70-7	1166	1125 [60]	MS, RI, STD	E	–	–	0.36±0.12	0.36±0.16	0.13±0.01	< LOQ
1,8-cineole	470-82-6	1173	1224 [57]	MS, RI, STD	T	–	–	0.43±0.18	–	–	–
beta-phellandrene	555-10-2	1175	1241 [57]	MS, RI	T	–	–	–	–	6.43±5.37	2.18±1.26
2-methylbutan-1-ol	137-32-6	1183	1212 [49]	MS, RI, STD	A	0.1±0.04	5.89±0.57	–	–	-	-
3-methylbutan-1-ol	123-51-3	1183	1215 [49]	MS, RI, STD	A	–	20.3±1.63	–	–	–	1.85±1.48
1-ethyl-3-methylbenzene	620-14-4	1196	1222 [61]	MS, RI, STD	H	–	–	–	–	0.05±0.01	–
1-ethyl-2-methylbenzene	611-14-3	1196	1257 [61]	MS, RI	H	–	–	2.17±1.48	0.27±0.1	0.25±0.10	–
Compounds^a^	CAS	RIcal^b^	RIref^c^	Identification^d^	Class^e^	*C. sativus* f	*F. culmorum* f	Non-infected roots g	*C. sativus*-infected roots g	*F. culmorum*-infected roots g	*C. sativus*+*F. culmorum* infected roots g
2-pentylfuran	3777-69-3	1210	1229 [62]	MS, RI, STD	F	-	-	19.51±10.80	1.64±0.80	1.35±0.98	< LOQ
gamma-terpinene	99-85-4	1215	1265 [57]	MS, RI, STD	T	-	-	-	-	1.45±1.02	0.42±0.22
Pentan-1-ol	71-41-0	1220	1255 [50]	MS, RI, STD	A	-	-	2.63±0.91	0.7±0.4	-	< LOQ
Pentyl propanoate	624-54-4	1223	1239 [49]	MS, RI	E	-	-	-	< LOQ	-	0.29±0.2
Octan-3-one	106-68-3	1230	1244 [63]	MS, RI, STD	K	-	6.95±1.57	-	3.14±0.58	18.03±15.94	7.02±3.45
3-methylbut-3-en-1-ol	763-32-6	1236	1264 [52]	MS, RI	A	< LOQ	–	–	–	–	–
para-cymene	99-87-6	1244	1277 [57]	MS, RI, STD	T	–	–	–	–	1.99±1.92	0.29±0.06
Heptan-4-ol	589-55-9	1261	1250 [64]	MS, RI	A	–	–	–	< LOQ	0.83±0.62	0.31±0.13
(E)-Pent-2-en-1-ol	1576-96-1	1272	1335 [49]	MS, RI, STD	A	–	–	0.54±0.21	-	-	-
2-(2-pentenyl)furan	70424-14-5	1273	-	MS, RI	F	–	–	0.50±0.35	-	-	-
Oct-1-en-3-one	4312-99-6	1275	1323 [57]	MS, RI, STD	K	–	–	–	< LOQ	1.12±0.65	0.69±0.28
5-methyl-hept-5-en-3-one	-	1282	-	MS	K	–	–	–	< LOQ	1.02±0.80	–
(Z)-Pent-2-en-1-ol	1576-95-0	1294	1313 [53]	MS, RI, STD	A	–	–	1.31±0.16	0.3±0.01	–	< LOQ
6-methyl-hept-5-en-2-one	110-93-0	1312	1319 [58]	MS, RI, STD	K	–	–	< LOQ	–	–	0.44±0.17
3-butenylbenzene	768-56-9	1329	-	MS	H	< LOQ	–	–	–	–	–
Hexan-1-ol	111-27-3	1330	1360 [57]	MS, RI, STD	A	–	–	4.47±0.58	0.22±0.04	-	< LOQ
Methyl octanoate	111-11-5	1362	1378 [47]	MS, RI, STD	E	–	–	0.63±0.34	< LOQ	0.25±0.21	< LOQ
Octan-3-ol	589-98-0	1373	1388 [63]	MS, RI, STD	A	–	–	–	< LOQ	1.58±1.30	0.82±0.28
(E)-Hex-2-en-1-ol	928-95-0	1376	1400 [65]	MS, RI, STD	A	–	–	1.14±0.33	–	–	–
1-methyl-2-(2-propenyl)-benzene	1587-04-8	1379	-	MS	H	< LOQ	–	–	–	–	–
2-methoxy-3-(1-methylethyl)-pyrazine	25773-40-4	1411	1427 [51]	MS, RI	O	–	–	–	–	–	< LOQ
Compounds^a^	CAS	RIcal^b^	RIref^c^	Identification^d^	Class^e^	*C. sativus* f	*F. culmorum* f	Non-infected roots g	*C. sativus*-infected roots g	*F. culmorum*-infected roots g	*C. sativus*+*F. culmorum* infected roots g
NI #3^h^	–	1412	–	–	Ae	–	–	0.2±0.07	–	–	–
Oct-1-en-3-ol	3391-86-4	1431	1438 [66]	MS, RI, STD	A	–	–	0.79±0.15	11.96±6.44	35.49±25.1	25.21±7.21
delta-elemene	20307-84-0	1444	1468 [67]	MS, RI	T	0.004±0.00	–	–	–	–	–
NI #4^h^	–	1444	–	–	F	–	–	0.40±0.13	–	0.05±0.01	–
NI #5 h	–	1445	–	–	Ae	–	–	–	< LOQ	< LOQ	0.37±0.03
2,6-dimethyloct-7-en-2-ol	53219-21-9	1457	1443 [68]	MS, RI	A	–	–	0.7±0.50	–	0.07±0.02	–
NI #6^h^	26456-76-8	1482	-	MS	Ae	–	–	0.24±0.07	12.69±3.36	5.88±3.87	11.15±1.97
2-methoxy-3-(1-methylpropyl)-pyrazine	24168-70-5	1485	1490 [51]	MS, RI, STD	O	–	–	–	–	–	< LOQ
2-butenylbenzene	1560-06-1	1488	-	MS	H	0.03±0.02	–	–	–	–	–
Pyrrole	109-97-7	1489	1509 [69]	MS, RI, STD	O	–	–	–	–	–	< LOQ
(E)-Non-2-enal	18829-56-6	1507	1528 [66]	MS, RI, STD	O	–	–	4.44±1.78	0.84±0.25	1.56±0.90	0.87±0.62
(+)-Sativene	3650-28-0	1508	1527 [70]	MS, RI, STD	T	82.45±17.41	–	–	46.73±9.59	–	11.05±3.44
NI #7 (sesquiterpene, MW = 204)^h^	-	1544	-	-	T	0.73±0.22	–	–	–	–	–
alpha-acoradiene	24048-44-0	1548	-	MS	T	-	–	–	–	1.73±0.94	0.2±0.02
Longifolene	475-20-7	1550	1574 [71]	MS, RI, STD	T	7.98±2.70	–	–	2.58±0.48	1.78±1.41	1.19±0.21
NI #8 (sesquiterpene, MW = 204)^h^	–	1555	-	–	T	–	–	–	–	0.75±0.47	< LOQ
NI #9 (sesquiterpene, MW = 204)^h^	–	1562	-	–	T	0.84±0.17	–	–	< LOQ	–	–
(2E,6Z)-nona-2,6-dienal	26370-28-5	1567	1605 [72]	MS, RI, STD	O	–	–	0.9±0.46	0.14±0.01	0.24±0.07	< LOQ
NI #10^h^	–	1571	–	–	NI	–	–	< LOQ	–	0.15±0.11	–
Epi-bicyclosesquiphellandrene	54324-03-7	1588	-	MS	T	–	–	–	–	3.55±1.08	0.36±0.05
Methyl non-2-enoate	111-79-5	1594	-	MS, STD	E	–	–	2.27±0.81	0.51±0.13	0.78±0.87	0.53±0.18
Compounds^a^	CAS	RIcal^b^	RIref^c^	Identification^d^	Class^e^	*C. sativus* f	*F. culmorum* f	Non-infected roots g	*C. sativus*-infected roots g	*F. culmorum*-infected roots g	*C. sativus*+*F. culmorum* infected roots g
NI #11^h^	–	1595	–	–	Ae	–	–	0.19±0.05	–	–	–
Methyl benzoate	93-58-3	1595	1635 [55]	MS, RI, STD	E	–	–	1.35±0.51	0.4±0.13	0.16±0.06	0.36±0.14
Widdrene	470-40-6	1609	1626 [71]	MS, RI	T	0.01±0.00	–	–	–	–	–
NI #12^h^	-	1631	-	-	NI	-	–	0.29±0.06	< LOQ	0.06±0.00	<LOQ
NI #13 (sesquiterpene, MW = 204)^h^	-	1633	-	-	T	0.40±0.14	–	–	–	–	–
Nonan-1-ol	143-08-8	1643	-	MS, STD	A	-	–	0.57±0.23	0.17±0.07	0.09±0.03	< LOQ
beta-acoradiene	28477-64-7	1645	-	MS	T	1.02±0.33	–	–	–	0.13±0.05	–
alpha-humulene	6753-98-6	1647	1663 [58]	MS, RI, STD	T	0.02±0.09	–	–	–	–	–
NI #14 (sesquiterpene, MW = 204)^h^	–	1651	–	MS	T	–	–	–	< LOQ	–	< LOQ
NI #15 (sesquiterpene, MW = 204)^h^	–	1654	–	–	T	0.62±0.22	–	–	–	–	–
cis-piperitol	16721-38-3	1657	1698 [58]	MS, RI	T	–	–	–	–	1.21±0.42	1.12±0.60
beta-terpineol	138-87-4	1659	1616 [71]	MS, RI, STD	T	–	–	1.03±0.17	< LOQ	< LOQ	< LOQ
(Z)-non-3-en-1-ol	10340-23-5	1666	1682 [73]	MS, RI	A	–	–	2.18±0.48	–	–	< LOQ
alpha-amorphene	483-75-0	1670	1691 [71]	MS, RI	T	0.04±0.01	–	–	–	–	–
NI #16 (sesquiterpene, MW = 204)^h^	–	1677	–	MS	T	0.32±0.12	–	–	–	–	–
alpha-terpineol	98-55-5	1691	1711 [48]	MS, RI	T	–	–	0.29±0.09	<LOQ	0.05±0.01	–
Epizonarene	41702-63-0	1697	1688 [74]	MS, RI	T	0.03±0.01	–	–	–	–	–
(E)-non-2-en-1-ol	31502-14-4	1700	1722 [75]	MS, RI, STD	A	–	–	16.78±5.46	0.29±0.05	0.15±0.02	< LOQ
3-methyl-6-propan-2-ylcyclohex-2-en-1-one	89-81-6	1704	1739 [71]	MS, RI	K	–	–	–	–	1.02±0.85	0.43±0.06
alpha-muurolene	10208-80-7	1711	1727 [71]	MS, RI	T	0.02±0.01	–	–	–	–	–
Compounds^a^	CAS	RIcal^b^	RIref^c^	Identification^d^	Class^e^	*C. sativus* f	*F. culmorum* f	Non-infected roots g	*C. sativus*-infected roots g	*F. culmorum*-infected roots g	*C. sativus*+*F. culmorum* infected roots g
trans-alpha-bisabolene	25532-79-0	1718	1740 [71]	MS, RI	T	0.33±0.15	–	–	–	–	–
Germacrene A	28387-44-2	1736	1737 [76]	MS, RI	T	1.93±0.66	–	–	< LOQ	–	< LOQ
(3E,6Z)-nona-3,6-dien-1-ol	56805-23-3	1739	1731 [54]	MS, RI	A	–	–	0.58±0.18	–	–	–
NI #17^h^	–	1748	–	–	NI	–	–	–	–	1.38±1.08	0.27±0.02
NI #18 (MW = 150)^h^	–	1748	–	–	H	–	–	–	< LOQ	–	0.32±0.05
(2E,6Z)-nona-2,6-dien-1-ol	28069-72-9	1748	1776 [77]	MS, RI, STD	A	–	–	8.14±3.54	< LOQ	0.04±0.00	-
n-dibutylformamide	761-65-9	1754	–	MS, STD	O	–	–	2.3±0.30	0.22±0.04	0.22±0.18	-
beta-sesquiphellandrene	20307-83-9	1759	1782 [58]	MS, RI	T	0.01±0.00	–	–	–	–	–
1-phenylbutan-1-one	495-40-9	1779	-	MS, STD	K	0.02±0.01	–	–	–	–	–
1-phenylethanol	98-85-1	1790	1795 [77]	MS, RI, STD	A	–	–	–	< LOQ	–	< LOQ
NI #19^h^	–	1846	–	–	NI	–	–	0.39±0.09	-	–	–
2-phenylethanol	60-12-8	1886	1859 [62]	MS, RI, STD	A	< LOQ	–	0.42±0.05	< LOQ	–	–
Tetradecanal	124-25-4	1910	1940 [77]	MS, RI, STD	O	–	–	0.9±0.57	–	0.33±0.15	–
1,3-benzothiazole	**95-16-9**	1930	1951 [78]	MS, RI, STD	O	–	–	0.93±0.41	< LOQ	0.16±0.03	< LOQ
5-pentyloxolan-2-one	104-61-0	2000	2063 [72]	MS, RI	K	–	–	0.9±0.48	< LOQ	0.1±0.07	–
NI #20^h^	**-**	2010	-	-	NI	0.5±0.10	–	–	< LOQ	–	–
6,10,14-trimethylpentadecan-2-one	**502-69-2**	2068	2131 [79]	MS, RI	K	–	–	0.55±0.16	–	–	–
Beyerene	3564-54-3	2108	–	MS	T	0.03±0.00	–	–	–	–	–
methyl hexadecanoate	**112-39-0**	2118	–	MS	E	–	–	< LOQ	–	0.04±0.01	< LOQ
methyl 2-aminobenzoate	134-20-3	2130	2260 [72]	MS, RI	E	–	–	–	–	–	< LOQ
Compounds^a^	CAS	RIcal^b^	RIref^c^	Identification^d^	Class^e^	*C. sativus* f	*F. culmorum* f	Non-infected roots g	*C. sativus*-infected roots g	*F. culmorum*-infected roots g	*C. sativus*+*F. culmorum* infected roots g
Isopimaradiene	1686-66-4	2144	-	MS	T	0.03±0.00	–	–	–	–	–
Isokaurene	5947-50-2	2159	-	MS	T	0.27±0.05	–	–	–	–	–
Chemical classes:											
Alcohols						0.1±0.04	53.39±4.42	4.049±0.92	13.98±1.02	38.25±3.87	28.49±1.85
Organic esters						0.01±0.00	–	11.94±0.63	14.26±1.03	3.87±0.46	29.6±0.39
Aromatic hydrocarbons						0.03±0.02	8.62±2.83	5.32±0.73	0.58±0.09	0.93±0.23	0.32±0.05
Ketones						0.02±0.01	7.64±1.02	4.85±0.29	3.87±0.36	23.6±2.76	10.35±0.72
Alkanes						–	30.4±22.63	1.45±0.36	0.66±0.04	0.4±0.21	< LOQ
Alkenes						0.01±0.00	–	0.63±0.06	13.75±1.94	6.15±1.94	12.84±0.74
Furanic compounds						–	–	20.41±3.76	1.64±0.80	1.4±0.49	< LOQ
Suphur compounds						–	–	1.2±0.26	0.75±0.43	0.35±0.26	0.15±0.06
Terpenoids						97.11±0.97	–	1.75±0.15	49.31±5.04	20.42±1.14	16.81±0.73
Others						–	–	9.47±0.70	1.2±0.1	2.51±0.27	0.99±0.34
						0.5±0.10	–	0.68±0.07	< LOQ	1.59±1.18	0.27±0.02

a Compounds listed from their order of elution in a VF-Wax polar capillary column (30 m×0.25 mm×0.25 µm).1

b Linear retention index calculated on a VF-Wax capillary column with a homologous series of n-alkanes (C7–C30).

c Linear retention index in literature.

d Identification proposal is indicated by the following: MS, identification by comparing EI mass spectrum with Wiley275, pal600k and NBS75K mass spectral database; RI, identification by retention indexes with literature data; STD, comparison with the retention times and mass spectra of available standards.

e Chemical classes: A: alcohol, E: organic ester, H: aromatic hydrocarbon, K: ketone, Aa: alkane, Ae: alkene, F: furanic compound, S: sulphur compound, T: terpenoid, O: other, NI: non identified.

f Mean percentage of VOCs emitted by 5 fungal cultures after 7 days, for an initial fungal inoculum of 2,000 conidia.

g Mean percentage emitted by 5 samples of 1 gram of dry barley roots after 7 days culture.

h Only the five major non-identified molecules (NI) of each treatment (> LOQ) were listed in the table.

< LOQ means that the mean quantity of the compound is below the limit of quantification (signal/noise ratio <3 at the same retention time).

Underscored figures are remarkable (see in the text).

The VOCs emitted by *F. culmorum* and *C. sativus* were quite different. Only 2-methylpropan-1-ol was common to the two fungi after 7 days (Table 1), and only methyl methanoate, 2-methylpropan-1-ol, 2-methylbutan-1-ol, and 3-methylbutan-1-ol were emitted by both the fungi over the whole period of analysis (Table S1).

**Figure 3:**
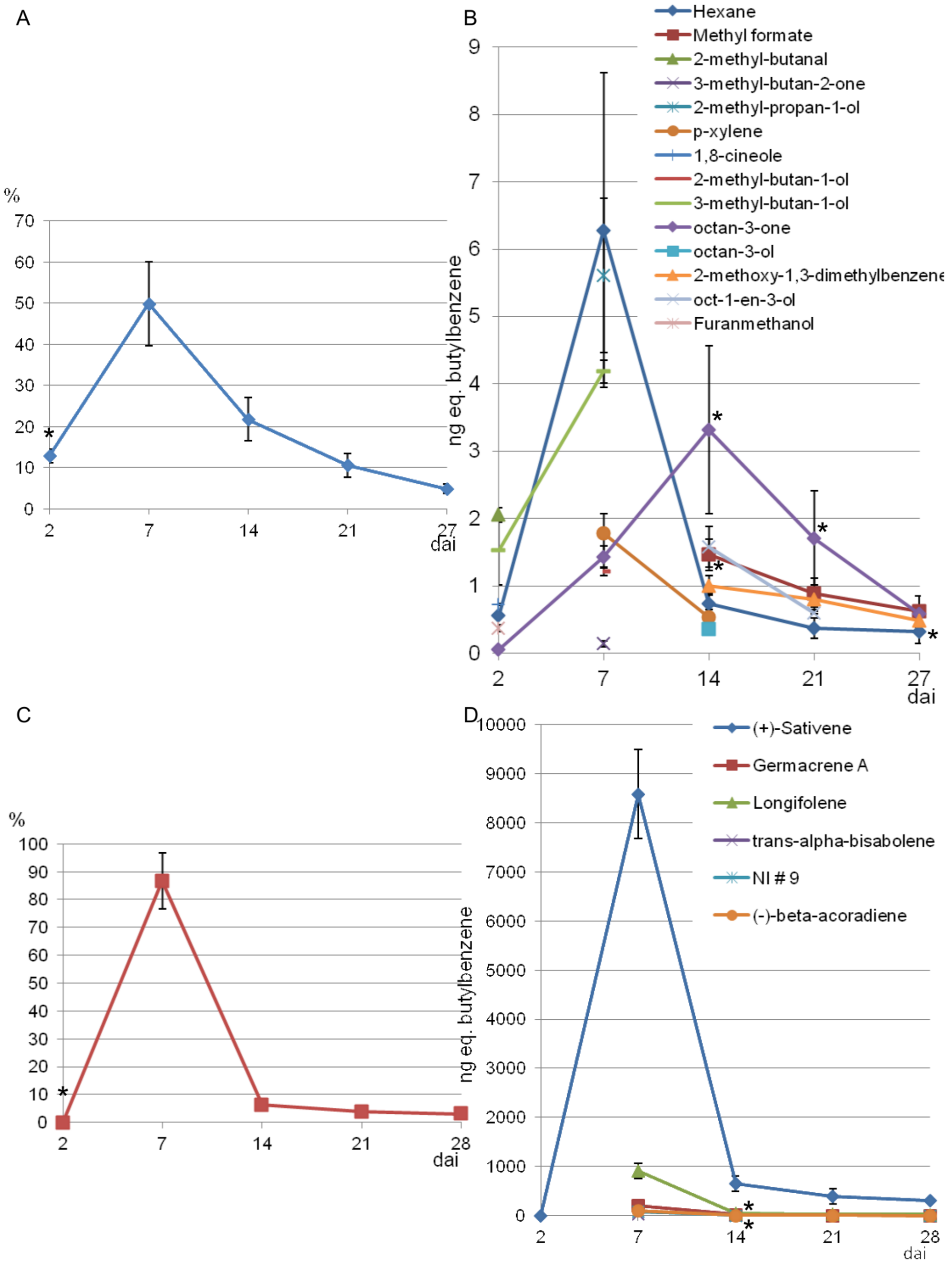
Time-course of VOC emission by *Fusarium culmorum* and *Cochliobolus sativus*. Time-course of total VOC emissions by *F. culmorum* (A) and *C. sativus* (C) and time-course of the emission of each VOC produced by *F. culmorum* (B) and of the 6 major VOCs produced by *C. sativus* (C and D) between 2 and 28 days culture. NI #9 means non-identified molecule number 9 (see Table 1). Stars (*) indicate a significant difference between the 5 replicates according to ANOVA (P<0.05).

Variations in fungal VOC emissions were monitored over time between 2 and 27 or 28 days culture on agar medium (Figure 3). ANOVAs (P<0.05) were carried out at each sampling time for all the main volatiles emitted by *C. sativus* and *F. culmorum*. These statistical tests compared the variances of the replicates 1, 2 and 3 and the replicates 4 and 5 which were analyzed at different periods of time. For F. culmorum, there were no significant differences between the replicates except for 3 volatiles: hexane after 27 days culture (p  = 0.017), methyl formate after 14 days culture (p  = 0.024) and octan-3-one after 14 (p  = 0.036) and 21 days culture (p  = 0.039). For *C. sativus*, no significant differences were observed except for 2 volatiles: Germacrène A (p  = 0.009) and longifolene (p  = 0.035) after 14 days culture.

All *F. culmorum* VOCs were first emitted within 14 days culture and the highest emission occurred after 7 days, when 50% of the relative quantity of VOCs was emitted (Figure 3, A and B). N-hexane, octan-3-one, 2-methylpropan-1-ol and 3-methylbutan-1-ol were the most abundant molecules (Table S1) : they represented 65% of the total relative quantity of VOCs emitted by *F. culmorum* in 27 days, even though 2-methylpropan-1-ol was emitted only punctually after 7 days culture. Some molecules were newly produced in relatively small quantities after 14 days; however total VOC emissions gradually decreased after 7 days.

Concerning *C. sativus*, VOCs were mainly produced after 7 days culture, when 87% of the relative quantity of VOCs was emitted. Sativene was by far the most abundant VOC emitted by *C. sativus*. It represented almost 83% of the total relative quantity emitted in 28 days. After the peak at 7 days, total VOC emissions decreased abruptly.

#### 1.2. VOCs emitted by infected barley roots

**Figure 4:**
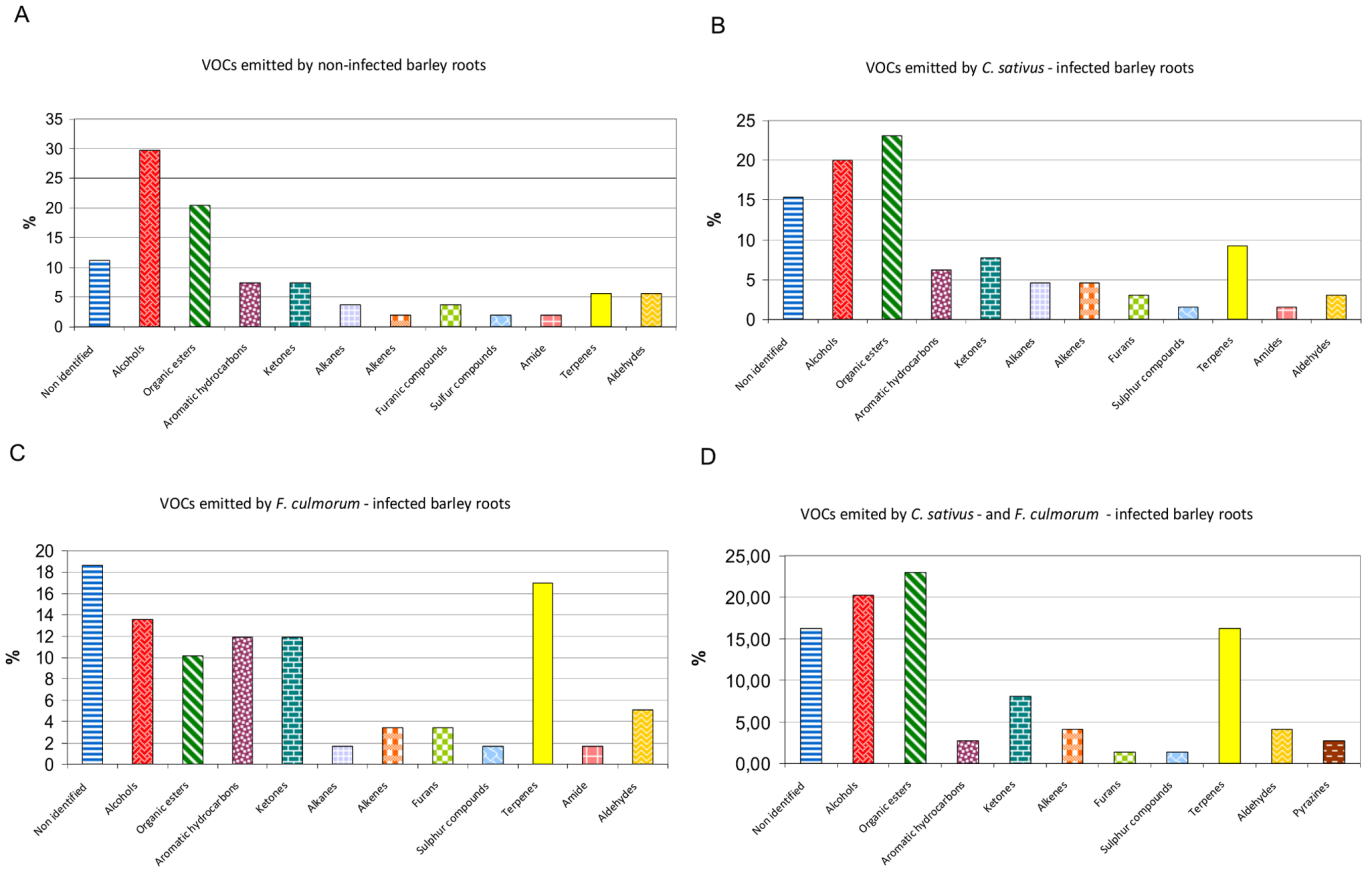
Chemical classes of the VOCs emitted by non-infected or infected barley roots. Classes of VOCs emitted by non-infected barley roots (A), by barley roots infected by *C. sativus* (B), infected by *F. culmorum* (C) or infected by the two fungi (D) after 7 days culture *in vitro* on Hoagland agar medium.

Seven days after inoculation, the fungal infections were well developed on barley roots and common root rot symptoms were visible on each plant. Volatiles were collected from *C. sativus-* and *F. culmorum-*infected roots and compared with volatiles from non-infected control plants. ANOVAs (P<0.05) were carried out to compare the variances of the 5 replicates of each treatment. There were no differences between the 5 replicates of healthy root analyses (p  = 0.343), analyses of roots infected by *C. sativus* (p  = 0.45), and analyses of roots infected by the two fungi (p  = 0.31). However, ANOVAs showed significant differences between the 5 replicates of analyses of roots infected by *F. culmorum* (p<0.0001). The fifth replicate was significantly different from the four other. GC-MS analyses of the collected volatiles showed major differences between the VOCs emitted by fungus-injured barley roots and the VOCs from non-infected roots. Around 54 VOCs (44 identified and 10 non-identified) were detected in non-infected roots analyses, 64 (52 identified and 12 non-identified) in *C. sativus*-infected roots, 60 (46 identified and 14 non-identified) in *F. culmorum*-infected roots, and 73 (59 identified and 15 non-identified) in roots infected by the two fungi (Table 1 and Figure 4). Major differences were observed between the VOCs released by non-infected and infected roots. New volatiles, not detected in fungal or non-infected root analyses, were observed when barley roots were attacked by a fungal pathogen. Twenty-three new VOCs (18 identified and 5 non-identified) were detected when the roots were attacked by *F. culmorum* (underscored figures in the column named “*F. culmorum* infected root” in Table 1), and 21 (16 identified and 5 non-identified) when they were attacked by *C. sativus* (underscored figures in the column named “*C. sativus* infected root” in Table 1). For *F. culmorum*-infected roots the newly released volatiles were terpenes (48%), ketones (17%), alcohols (9%), alkanes (9%), alkenes (9%), aromatic hydrocarbons (4%) and non-identified volatiles (4%). For *C. sativus*-infected roots new VOCs were organic esters (27%), alcohols (23%), ketones (18%), alkenes (14%), alkanes (9%), terpenes (6%), and aromatic hydrocarbons (6%). Terpenes emitted by non-infected barley roots were 1,8-cineole, α-terpineol and β-terpineol; they represented only 1,75% of the total relative VOC quantity. Their production increased dramatically when the roots were attacked by *F. culmorum*. After 7 days culture, the major newly released terpenes were β-phellandrene, epi-bicyclosesquiphellandrene, para-cymene, and longifolene (6.43%, 3.55%, 1.99%, and 1.78% of the total relative VOC quantity, respectively). *C. sativus*-infected roots released rather new organic esters and ketones : methyl propanoate, methyl methanoate and methyl acrylate (3.43%, 3.36%, 1.75% of the total relative VOC quantity, respectively). *C. sativus*-infected roots also released octan-3-one and octa-1,3-diene in high relative amounts (3.14% and 1.06% of the total relative VOC quantity, respectively). Compared with non-infected roots, all infected roots samples showed remarkable increases in oct-1-en-3-ol and NI #6. Altogether, non-infected roots released higher amounts of VOCs than infected roots but some VOCs showed surprisingly low levels compared with infected roots (*e.g.* 2-pentylfuran, (E)-non-2-en-1-ol or (2E, 6Z)-2,6-nonadien-1-ol). Moreover, ten volatiles were only emitted by non-infected roots (*i.e.* ethyl acetate, 1,8-cineole, (E)-pent-2-en-1-ol, 2-(2-pentenyl)furan, (E)-hex-2-en-1-ol, NI #3, NI #11, (3E, 6Z)-3,6-nonadien-1-ol, NI #19, and 6,10,14-trimethyl-2-pentadecanone) (underscored figures in the column named “Non-infected roots” in Table 1).

When barley roots were attacked by both *F. culmorum* and *C. sativus*, they generally emitted the same VOCs as detected in the trials with fungi alone and with mono-infected barley roots. But five compounds were newly released in trace amounts when barley was infected by both fungi: (E)-hex-2-enal, 2-methyoxy-3-(1-methylethyl)-pyrazine, 2-methyoxy-3-(1-methylpropyl)-pyrazine, pyrrole and methyl 2-aminobenzoate (underscored figures in the column named “*C. sativus*+*F. culmorum* infected roots in Table 1).

In addition to the compounds mentioned in Table 1, one non-identified alcohol representing 0.19% of the total relative VOC quantity was detected in non-infected barley roots. Six non-identified VOCs, including two terpenoids, were detected in trace amounts in *C. sativus*-infected barley roots, five non-identified VOCs representing 0.15% of the total relative VOC quantity were detected in *F. culmorum*-infected barley roots, and seven non-identified VOCs representing 0.18% of the total relative VOC quantity were detected in *C. sativus*- and-*F. culmorum*-infected barley roots.

### 2. *In vitro* Co-culture of Fungi and Barley

#### 2.1. Effects of the blend of VOCs from non-infected barley roots on pathogenic fungi

The effect of the volatiles released by non-infected barley roots on *F. culmorum* and *C. sativus* was assessed by cultivating the two organisms *in vitro* in a shared atmosphere. In the 3 independent trials, barley developed well and, after 9 days co-culture, aerial parts were between 5 and 20 cm high, whereas roots were between 3 and 15 cm long with a few secondary roots. All the plants reached approximately the 12^th^ Zadoks’ stage [22]. Fungal growth was never totally inhibited. As *F. culmorum* grew faster than *C. sativus* on water agar medium, the diameter of *F. culmorum* was measured only 144 or 168 hai, until the fungus colonized the whole Petri dish. The diameter of *C. sativus* was measured until 192 hai.

**Figure 5:**
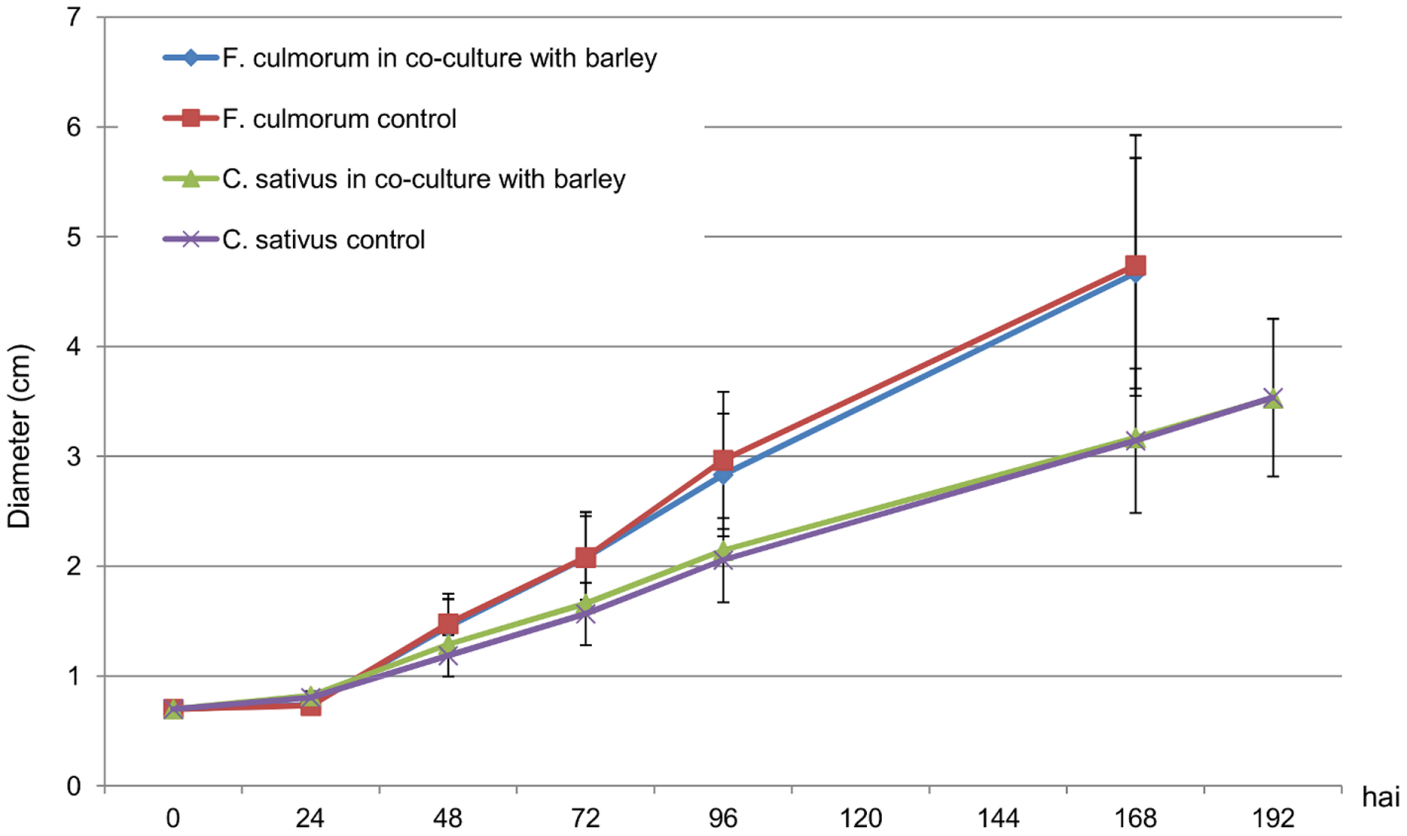
Effect of VOCs from non-infected barley roots on *Fusarium culmorum* and *Cochliobolus sativus* growth. Time-course of *F. culmorum* growth when cultivated alone (red squares) or in the presence of VOCs from non-infected barley roots (blue diamonds) and of *C. sativus* growth when cultivated alone (purple crosses) or in the presence of VOCs from non-infected barley roots (green triangles).

ANOVAs showed that no significant change occurred in fungal growth when the fungi were cultivated in the presence of VOCs from non-infected barley roots (Figure 5).

#### 2.2. Effects of the blend of VOCs from infected barley roots on pathogenic fungi

The effects of the volatiles released by infected barley roots on *F. culmorum* and *C. sativus* were assessed by cultivating each fungus *in vitro* in an atmosphere shared with barley roots infected by *F. culmorum* or *C. sativus*. Fungal growth was compared with a control where each fungus was grown in a plate where only volatiles were exchanged with non-infected barley roots within the head-space.

**Figure 6:**
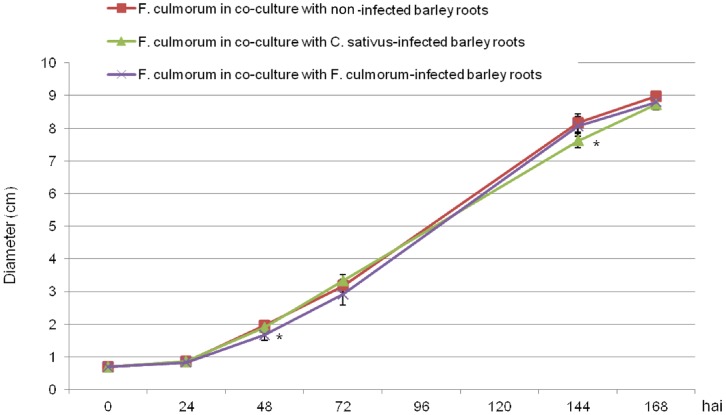
Effect of VOCs from non-infected or infected barley roots on the growth of *Fusarium culmorum*. Time-course of *F. culmorum* growth when cultivated in the presence of VOCs from non-infected barley roots (red squares), from *C. sativus*-infected roots (light green triangles) or from *F. culmorum*-infected roots (purple crosses). Stars (*) indicate a significant difference from the control according to Dunnett’s test (*α* <0.05).

The diameter of *F. culmorum* grown with non-infected barley roots was slightly larger than when it was grown with infected roots (Figure 6). However, significant differences were observed only punctually 48 hrs after infection for *F. culmorum*-infected barley roots (p  = 0.029) and 144 hrs after infection for *C. sativus*-infected barley roots (p  = 0.034) (Figure 6). Therefore, we cannot conclude that the VOCs released by infected barley roots had a long-term effect on *F. culmorum* growth.

**Figure 7:**
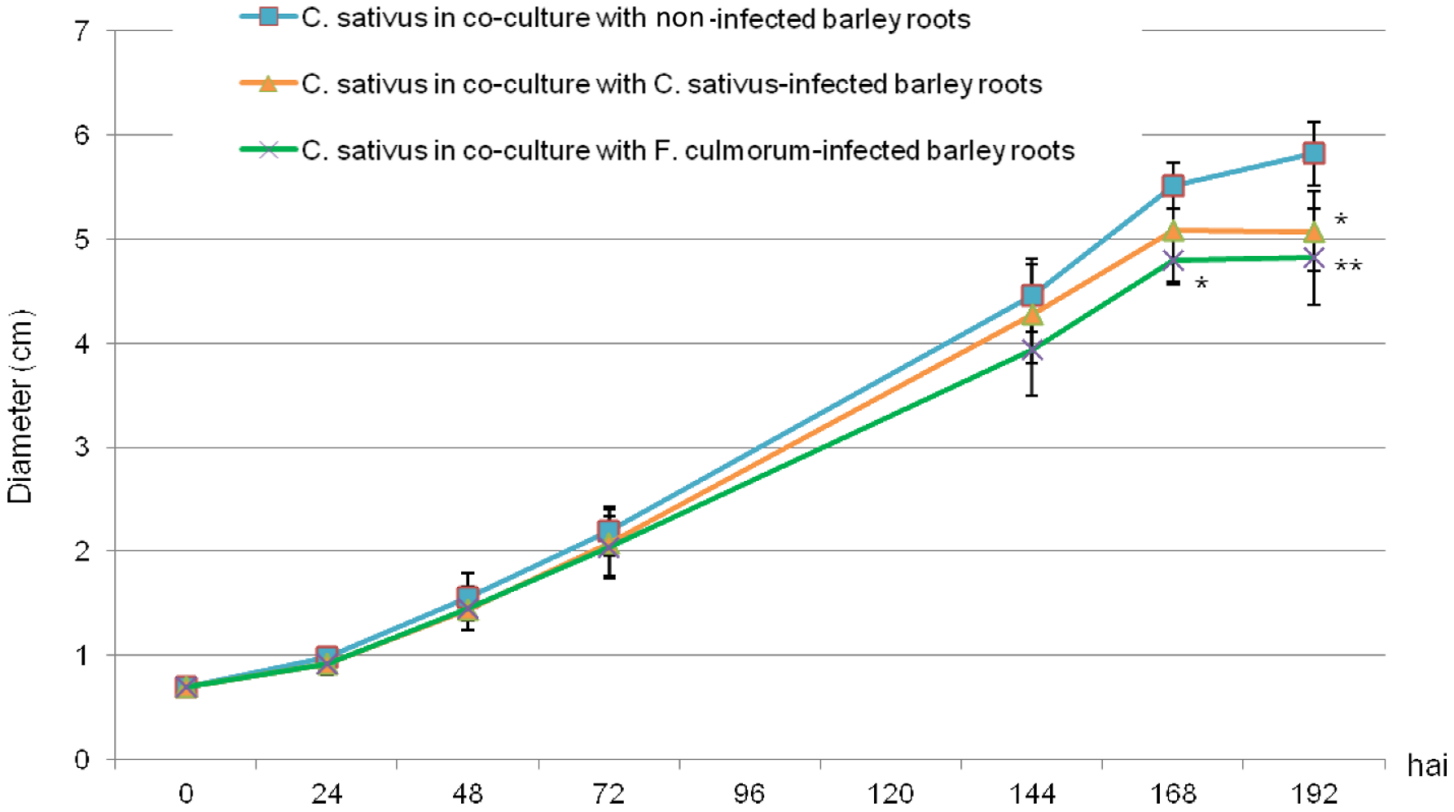
Effect of VOCs from non-infected or infected barley roots on the growth of *Cochliobolus sativus*. Time-course of *C. sativus* growth when cultivated in the presence of VOCs from non-infected barley roots (blue squares), from *C. sativus*-infected roots (orange triangles) or from *F. culmorum*-infected roots (dark green crosses). Stars (*/**) indicate a significant difference from the control according to Dunnett’s test (α <0.05 or α <0.01, respectively).

According to Dunnett’s tests, the diameter of *C. sativus* was significantly smaller when the fungus shared the atmosphere of *F. culmorum*-infected barley roots from 168 hrs after infection (p  = 0.032) and fungal growth was decreased by more than 13% 168 hai and up to 17% 192 hai (Figure 7).

In an atmosphere containing VOCs from *C. sativus*-infected roots, *C. sativus* growth was significantly decreased (p  = 0.005) by about 13% 192 hrs after infection (Figure 7). Therefore, the blend of VOCs released by infected barley roots significantly decreased *C. sativus* growth.

#### 2.3. Effects of the blend of VOCs from pathogenic fungi on barley

The effect of fungal VOCs on barley development was studied in similar experimental devices to those previously described. In each Petri dish, two barley seeds were grown in an atmosphere shared with *F. culmorum*, *C. sativus* or a control. Thus, for each treatment, 20 barley plants were measured. Non-germinated barley seeds were not taken into account in the statistical analyses. Throughout the four trials, 69 barley plants were analyzed following ‘*F. culmorum*’ treatment, 64 were analyzed following ‘*C. sativus*’ treatment, and 60 were analyzed for the control treatment.

**Figure 8:**
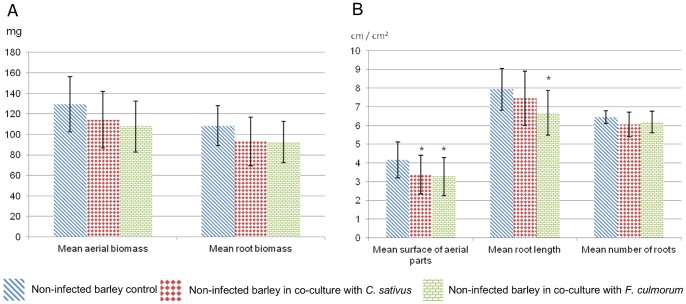
Effect of fungal VOCs on barley growth. Biomass of aerial parts and roots (A), surface of aerial parts, mean root length and number (B) of barley seedlings grown in the absence of fungal VOCs (blue diagonals) or in the presence of VOCs emitted by *C. sativus* (red diamonds) or *F. culmorum* (green bricks). Stars (*) indicate a significant difference from the control according to Dunnett’s test (α <0.05).

The statistical tests showed no significant difference between treatments and control for the biomass of aerial parts (p  = 0.065), of belowground parts (p  = 0.069) and for the number of roots (p  = 0.12) (Figure 8). On the other hand, ANOVAs and Dunnett’s tests showed that the VOCs emitted by *F. culmorum* significantly decreased leaf surface by 21.5% (p  = 0.027) and mean root length by 15% (p  = 0.019). The VOCs emitted by *C. sativus* decreased leaf surface by 19%. This allows us to conclude that fungi can affect plant growth by emitting VOCs.

## Discussion

The aim of this study was to characterize the volatile interactions between non-infected or infected barley roots and two pathogenic fungi, *C. sativus* and *F. culmorum* that cause common root rot on cereals. To our knowledge, it is the first paper that deals with below-ground volatile interactions between plants and pathogenic fungi. Our results show that a pathogenic fungal infection on barley roots induces VOC production and that the blend of these VOCs slowed down the growth of our pathogens, especially *C. sativus*. Therefore those molecules or their induction are promising methods for controlling common root rot on barley crops.

To date, a database of volatiles emitted by microorganisms (DOVE-MO) compiles approximately 300 bacteria and fungi described as VOC producers [23]. *C. sativus* and *F. culmorum* are not listed in it, and to our knowledge this is the first time that the VOCs from those two pathogenic fungi have been precisely described.

There are four sources of variation of volatile emission: the production of biological material, the sampling with an SPME fibre, the chromatographic separation, the mass spectrometer response. As explained previously, all the needed precautions were taken and the machines were properly calibrated before each analysis. Statistical tests were carried out to check that the 5 replicates of each treatment that were analyzed at two different dates were comparable. The tests showed that there was no difference between the 5 replicates of each treatment except for fungal analyses at 2 days culture and for the analyses of roots infected by *F. culmorum*. The fifth replicate analyzed on the 1^st^ February 2012 was different from the four others replicates that were analyzed on the 19 December 2011 (n°1, 2 and 3) and the 1^st^ February 2012 (n°4). This shows that biological variations are more important than the date of the analyses. Moreover, the VOCs that were not detected at the two analysis dates were not considered in the results. Only the molecules with a stable emission in time were taken into account. Thus, the 5 replicates of each treatment can be compared even if they were not analyzed at the same time and the volatile emission by the fungi can be compared with the emissions of volatiles by infected roots.

The analyses of fungal VOCs show that 2 pathogenic fungi can emit very different molecules. Only 5 metabolites were released by both fungi in 27 days. According to several studies, 2-methylpropan-1-ol, 3-methylbutan-1-ol and oct-1-en-3-ol are commonly produced by fungi [24]–[26]. Other volatiles are more specifically emitted. For example, sativene was first isolated from *C. sativus* and is released in high amounts by that fungus [27]. *F. culmorum* rather seems to emit VOCs commonly produced by other fungal species. The diversity of detected volatiles depends on the number and the sensitivity of the techniques set up [23]. Moreover, VOC amounts and nature depend on nutrient availability and culture conditions, but the main VOCs generally remain unchanged [28]–[30]. Our results show a maximal emission of fungal VOCs after 7 days culture in vials. This period corresponds approximately to the time needed for the fungus to colonize the whole agar surface in the vials. Fungal growth rate probably modifies VOC emissions by fungi.

Many papers report analyses of the VOCs emitted by aerial parts or seedlings of cereals, and the induction of new aerial volatiles during a fungal infection of barley roots [31]–[33]. However, this study investigates for the first time the VOCs of cereal roots and demonstrates that a fungal attack on barley induces below-ground volatiles emission. The observed variations suggest that the interaction between barley roots and pathogenic fungi modifies the metabolism of both barley and fungi by causing them to switch on/off the emission of certain VOCs. Since different volatiles are induced by *C. sativus* and *F. culmorum*, the molecular pathways induced by fungal infections can be expected to differ according to the pathogen. In our experimental conditions, the origin of the newly produced VOCs remains uncertain. They can be released either by barley roots or by fungi. The most surprising change in volatile profile was the increase in terpene production after *F. culmorum* infection. Several studies report the induction of volatile production, especially of terpenoid volatiles, involved in plant defence systems, before, during or after a biotic or an abiotic stress. Literature suggests that the newly emitted VOCs are released by plant roots rather than by fungi as the most common volatile signals involved in direct and indirect defences of plant aerial parts include metabolites of the lipoxygenase (LOX) pathway, the shikimic acid pathway, and products of the terpenoid pathway (monoterpenes, sesquiterpenes, homoterpenes). Some genes inducing VOC emission have also been recently isolated from plants [34], [35]. It is attractive to think that the production of terpenoids released in the soil could depend on the same molecular mechanisms as in aerial parts. Molecular and genetic analyses are needed to trace the origin of those VOCs and to determine the metabolic mechanisms that underlie the variations in volatile emissions, especially the increase in terpene emissions.

Two experimental setups for co-cultivation were designed to match the observation needs. In the device made to study the effects of the VOCs of barley roots on fungi, the fungi were grown on water agar in large Petri dishes to not influence their growth with supplementary nutrients or dish wall. In the device made to study the effects of the fungal VOCs on barley, fungi were grown on PDA in small Petri dishes to favour their growth and the emission of VOCs and to give more space to barley to grow.

In our experiments, volatiles from non-infected barley roots and from *F. culmorum*-infected roots did not influence the growth of the pathogenic fungi. However, *C. sativus* growth was significantly affected by the blend of VOC released by *C. sativus*-infected roots. The decreased fungal growth we observed might result from a limitation of enzyme synthesis rather than an inhibition of enzyme activity [36]. The most current examples of volatile induction for plant defence concern herbivore attacks [37]. But our results suggest, like other studies, that the VOCs emitted *de novo* during a fungal attack play a role in the induced systemic resistance of plants against pathogenic fungi [34]. Plant volatiles can also affect fungal reproduction and cellular development [38]. This study stresses the need for assessing the effects of the VOCs released by infected barley roots on *C. sativus* and *F. culmorum* sporulation and cellular morphology. Furthermore, the effects of some major induced volatiles, taken independently, on the growth and cellular development of pathogenic fungi also need to be investigated.

From another point of view, the smaller barley leaf surface resulting from the VOCs emitted by *C. sativus* and *F. culmorum* and the shorter mean root length resulting from the volatiles released by *F. culmorum* suggest that pathogenic fungi interact with plants through volatiles. This phenomenon could be part of the “pathogenic strategy” of fungi. VOCs emitted by pathogenic fungi could weaken plants before any physical contact between the two organisms [17], [18]. Some studies show that *C. sativus* produces sesquiterpenoid toxins that are synthesized from farnesol. We detected cyclosativene and (-)-sativene, precursors of helminthosporol [39], [40]. Helminthosporol, prehelminthosporal and helminthosporal are crop-destroying toxins that inhibit the respiration of roots and coleoptiles and affect the cellular metabolism of different varieties of barley and wheat [15], [41]. However, other studies indicate that helminthosporol and helminthosporic acid activate α-amylase production as gibberellin does and thus promote seedling growth [42]. Concerning *F. culmorum*, the sesquiterpene-derived toxins trichothecenes (*i.e.* deoxynivalenol, nivalenol and zearalenone) are potent inhibitors of protein synthesis and are hypothesized to inhibit the activation of defence response genes [18]. Trichodiene, a volatile marker of trichothecene biosynthesis, was not detected in our analyses [43], [44]. We can therefore suggest that the pathogenicity of our *F. culmorum* strain toward barley was not due to mycotoxins but to other VOCs emitted by the fungus.

Our study let think that the bitrophic rhizospheric interaction between one plant and one or two pathogenic fungi involves fungal VOCs as pathogenic factors and plant VOCs as defence agents. However, interactions that take place in the rhizosphere include a much larger number of organisms. The rhizosphere is a very complex and competitive environment that includes detrimental or beneficial bacteria or fungi, insects, plants and viruses, and is submitted to all the local abiotic parameters. Thus volatile interactions rarely concern only two organisms. Inside the soil, the diffusion of the volatile molecules involved in those interactions depends on the chemical properties of the volatiles themselves and on the physico-chemical properties of the soil. Diffusion, adsorption and biodegradation phenomena determine volatile distribution below-ground [23]. Thus the effects of volatiles observed *in vitro* are most probably different from those observed *in vivo*, especially as soils can have their own intrinsic fungistatic properties [45].

In conclusion, the volatiles produced *de novo* during the interaction between barley and pathogenic fungi represent new opportunities for biological control methods. The effects of those VOCs observed *in vitro* are promising. Control methods could consist in directly applying synthesized volatiles to cereal fields or in eliciting volatile production to activate the induced defence systems of plants. However, the induction mechanisms at the metabolic and genetic levels and the behaviour of protective volatiles in natural environments remain to be characterized in order to develop an efficient environment-friendly method for cereal protection.

## Supporting Information

Table S1 VOCs emitted by the pathogenic fungi *Fusarium culmorum* and by *Cochliobolus sativus* after 2, 7, 14, 21, and 27 or 28 days.
(DOC)
