# Supplementary material for: Volatile Compound-Mediated Interactions between Barley and Pathogenic Fungi in the Soil
Source: PLoS One. 2013 Jun 20;8(6):e66805. doi: 10.1371/journal.pone.0066805 (PMC3688563; doi:10.1371/journal.pone.0066805)
Supplement: Table S1 — VOCs emitted by the pathogenic fungi Fusarium culmorum and by Cochliobolus sativus after 2, 7, 14, 21, and 27 or 28 days. (DOC) [file pone.0066805.s001.doc]

| Supplementary data: VOCs emitted by the pathogenic fungi *Fusarium culmorum* and by *Cochliobolus sativus* after 2, 7, 14, 21, and 27 or 28 days | | | | | | | | | | | | | | | | |
| --- | --- | --- | --- | --- | --- | --- | --- | --- | --- | --- | --- | --- | --- | --- | --- | --- |
|  |  |  |  |  |  | Emitted by | | | | | | | | | | |
|  |  |  |  |  |  | *C. sativus* ^f^ | | | | | *F. culmorum* ^f^ | | | | | |
| Compounds^a^ | CAS | RIcal^b^ | RIref^c^ | Identification^d^ | Class^e^ | 2 days | 7 days | 14 days | 21 days | 28 days | 2 days | | 7 days | 14 days | 21 days | 27 days |
| Hexane | 110-54-3 | 600 | 600 | MS, RI, STD | Aa | - | - | - | - | - | 10.62±1.10 | | 30.4±22.63 | 8.17±3.36 | 8.50±6.75 | 15.93±16.86 |
| 2-methylbuta-1,3-diene | 78-79-5 | 665 | - | MS | Ae | - | 0.01 ± 0.00 | - | - | - | - | | - | - | - | - |
| Heptane | 142-82-5 | 700 | 700 | MS, RI, STD | Aa | - | - | 0.06±0.02 | - | - |  | |  |  |  |  |
| Methyl methanoate | 107-31-3 | 736 | 682^(46)^ | MS, RI | E | - | - | 0.02±0.00 | 0.06±0.01 | 0.07±0.01 | - | | - | 16.25±5.15 | 20.19±10.70 | 30.66±23.25 |
| 2-methylbutanal | 96-17-3 | 840 | 926 ^(56)^ | MS, RI | O | - | - | - | - | - | 38.77±3.91 | | - | - | - | - |
| 3-methylbutan-2-one | 563-80-4 | 918 | - | MS, STD | K | - | - | - | - | - | - | | 0.69±0.46 | - | < LOQ | < LOQ |
| 2-methylpropyl formate | 542-55-2 | 953 | - | MS, STD | E | - | 0.01±0.003 | - | - | - | - | | - | - | - | - |
| 2-methylbut-3-en-2-ol | 115-18-4 | 1020 | 1036^(53)^ | MS, RI, STD | A | < LOQ | < LOQ | < LOQ | < LOQ | < LOQ | - | | - | - | - | - |
| 2-methylpropan-1-ol | 78-83-1 | 1103 | 1108^(55)^ | MS, RI | A | - | < LOQ | 0.03±0.01 | < LOQ | - | - | | 27.2±11.07 | - | - | - |
| para-xylene | 106-42-3 | 1109 | 1127^(49)^ | MS, RI, STD | H | - | - | - | - | - | < LOQ | | 8.62±2.83 | 5.96±2.46 | < LOQ | < LOQ |
| 1,8-cineole | 470-82-6 | 1173 | 1224^(57)^ | MS, RI, STD | T | - | - | - | - | - | 13.68±0.61 | | - | - | - | - |
| 2-methylbutan-1-ol | 137-32-6 | 1183 | 1212^(49)^ | MS, RI, STD | A | - | 0.1±0.04 | 0.05±0.01 | < LOQ | < LOQ | - | | 5.89±0.57 | - | - | - |
| 3-methylbutan-1-ol | 123-51-3 | 1183 | 1215^(49)^ | MS, RI, STD | A | 87.30±53.18 | - | - | - | - | 28.85±19.31 | | 20.3±1.63 | - | - | - |
| Octan-3-one | 106-68-3 | 1230 | 1244^(63)^ | MS, RI, STD | K | - | - | - | - | - | 1.07±0.00 | | 6.95±1.57 | 36.86±27.58 | 39.09±32.04 | 29.31±0.76 |
| 3-methylbut-3-en-1-ol | 763-32-6 | 1236 | 1264^(52)^ | MS, RI | A | - | < LOQ | - | - | - | - | | - | - | - | - |
| 6-methyl-hept-5-en-2-one | 110-93-0 | 1312 | 1319^(58)^ | MS, RI, STD | K | < LOQ | - | - | - | - | - | | - | - | - | - |
|  |  | RIcal^b^ | RIref^c^ | Identification^d^ | Class^e^ | *C. sativus* ^f^ | | | | | *F. culmorum* ^f^ | | | | | |
| Compounds^a^ | CAS |  |  |  |  | 2 days | 7 days | 14 days | 21 days | 28 days | 2 days | 7 days | | 14 days | 21 days | 27 days |
| 3-butenylbenzene | 768-56-9 | 1329 | - | MS | H | - | < LOQ | < LOQ | - | - | - | - | | - | - | - |
| Hexan-1-ol | 111-27-3 | 1330 | 1360^(57)^ | MS, RI, STD | A | < LOQ | - | - | - | - | - | - | | - | - | - |
| Dimethyltrisulfide | 3658-80-8 | 1347 | 1377 ^(56)^ | MS, RI | O | < LOQ | - | - | - | - | - | - | | - | - | - |
| Octan-3-ol | 589-98-0 | 1373 | 1388^(63)^ | MS, RI, STD | A | - | - | - | - | - | - | - | | 3.99±0.60 | - | < LOQ |
| 1-methyl-2-(2-propenyl)-benzene | 1587-04-8 | 1379 | - | MS | H | - | < LOQ | - | - | - | - | - | | - | - | - |
| 2-methyl-1,3-dimethylbenzene | 1004-66-6 | 1396 | - | MS, STD | H | - | - | - | - | - | - | < LOQ | | 11.16±3.20 | 18.43±5.48 | 24.10±1.72 |
| Oct-1-en-3-ol | 3391-86-4 | 1431 | 1438^(66)^ | MS, RI, STD | A | < LOQ | - | < LOQ | 0.09±0.01 | < LOQ | - | - | | - | - | - |
| 3-methylsulfanylpropanal | 3268-49-3 | 1435 | 1463 ^(66)^ | MS, RI | O | < LOQ | - | - | - | - | - | - | | - | - | - |
| delta-elemene | 20307-84-0 | 1444 | 1468^(67)^ | MS, RI | T | - | 0.004±0.00 | < LOQ | - | < LOQ | - | - | | - | - | - |
| alpha-ylangene | 14912-44-8 | 1455 | 1491 ^(71)^ | MS, RI | T | - | - | < LOQ | < LOQ | < LOQ | - | - | | - | - | - |
| Cyclosativene | 22469-52-9 | 1466 | 1473 ^(70)^ | MS, RI | T | - | - | < LOQ | < LOQ | < LOQ | - | - | | - | - | - |
| 2-butenylbenzene | 1560-06-1 | 1488 | - | MS | H | - | 0.03±0.02 | < LOQ | < LOQ | - | - | - | | - | - | - |
| Pentadecane | 629-62-9 | 1500 | 1500 | MS, RI | Aa | < LOQ | - | < LOQ | - | - | - | - | | - | - | - |
| (+)-Sativene | 3650-28-0 | 1508 | 1527^(70)^ | MS, RI, STD | T | 12.70±10.64 | 82.45±17.41 | 84.17±40.16 | 85.34±67.29 | 81.93±9.79 | - | - | | - | - | - |
| NI #7 (sesquiterpene, MW = 204) ^g^ | - | 1544 | - | - | T | - | 0.73 ± 0.22 | 5.19±2.25 | 6.18±5.27 | 6.31±0.48 | - | - | | - | - | - |
| Longifolene | 475-20-7 | 1550 | 1574^(71)^ | MS, RI, STD | T | - | 7.98±2.70 | 1.05±1.13 | - | - | - | - | | - | - | - |
| NI #9 (sesquiterpene, MW=204) ^g^ | - | 1562 | - | - | T | - | 0.84±0.17 | 0.73±0.27 | < LOQ | 0.62±0.07 | - | - | | - | - | - |
| Widdrene | 470-40-6 | 1609 | 1626^(71)^ | MS, RI | T | - | 0.01±0.00 | - | - | - | - | - | | - | - | - |
| 2-furanmethanol | 98-00-0 | 1617 | 1686 ^(52)^ | MS, RI | A | - | - | - | - | - | 7.00±1.90 | - | | - | - | - |
| NI #14 (sesquiterpene, MW=204) ^g^ | - | 1633 | - | - | T | - | 0.40±0.14 | < LOQ | - | 0.16±0.02 | - | - | | - | - | - |
|  |  | RIcal^b^ | RIref^c^ | Identification^d^ | Class^e^ | *C. sativus* ^f^ | | | | | *F. culmorum* ^f^ | | | | | |
| Compounds^a^ | CAS |  |  |  |  | 2 days | 7 days | 14 days | 21 days | 28 days | 2 days | 7 days | | 14 days | 21 days | 27 days |
| beta-acoradiene | 28477-64-7 | 1645 | - | MS | T | - | 1.02±0.33 | 0.54±0.09 | 0.87±0.81 | 0.74±0.06 | - | - | | - | - | - |
| alpha-humulene | 6753-98-6 | 1647 | 1663^(58)^ | MS, RI, STD | T | - | 0.02±0.09 | < LOQ | - | - | - | - | | - | - | - |
| NI #21 (sesquiterpene, MW=204) ^g^ | - | 1651 | - | - | T | - | - | 0.30±0.37 | - | - | - | - | | - | - | - |
| NI #16 (sesquiterpene, MW=204) ^g^ | - | 1654 | - | - | T | - | 0.62±0.22 | 0.15±0.04 | 0.31±0.12 | 0.16±0.02 | - | - | | - | - | - |
| alpha-amorphene | 483-75-0 | 1670 | 1691^(71)^ | MS, RI | T | - | 0.04±0.01 | < LOQ | < LOQ | < LOQ | - | - | | - | - | - |
| NI #17 (sesquiterpene, MW=204) ^g^ | - | 1677 | - | MS | T | - | 0.32±0.12 | 0.17±0.07 | 0.19±0.10 | - | - | - | | - | - | - |
| Epizonarene | 41702-63-0 | 1697 | 1688^(74)^ | MS, RI | T | - | 0.03±0.01 | < LOQ | < LOQ | - | - | - | | - | - | - |
| NI #22 (sesquiterpene, MW=204) ^g^ | - | 1700 | - | MS | T | - | 0.07±0.02 | 0.07±0.02 | < LOQ | < LOQ | - | - | | - | - | - |
| alpha-muurolene | 10208-80-7 | 1711 | 1727^(71)^ | MS, RI | T | - | 0.02±0.01 | < LOQ | < LOQ | < LOQ | - | - | | - | - | - |
| trans-alpha-bisabolene | 25532-79-0 | 1718 | 1740^(71)^ | MS, RI | T | - | 0.33±0.15 | < LOQ | < LOQ | < LOQ | - | - | | - | - | - |
| Germacrene A | 28387-44-2 | 1736 | 1737^(76)^ | MS, RI | T | - | 1.93±0.66 | 3.13±4.94 | 1.12±0.88 | 1.03±0.22 | - | - | | - | - | - |
| beta-sesquiphellandrene | 20307-83-9 | 1759 | 1782^(58)^ | MS, RI | T | - | 0.01±0.00 | - | - | - | - | - | | - | - | - |
| 1-phenylbutan-1-one | 495-40-9 | 1779 | - | MS, STD | K | - | 0.02±0.01 | - | - | - | - | - | | - | - | - |
| 1-phenylethanol | 98-85-1 | 1790 | 1795^(77)^ | MS, RI, STD | A | < LOQ | - | - | - | - | - | - | | - | - | - |
| 2-phenylethanol | 60-12-8 | 1886 | 1859 ^(62)^ | MS, RI, STD | A | < LOQ | < LOQ | - | - | - | - | - | | - | - | - |
| NI #23 ^g^ | - | 1895 | - | - | NI | - | 0.17±0.11 | 0.21±0.03 | 0.36±0.19 | 0.70±0.14 | - | - | | - | - | - |
| NI #24^g^ | - | 2010 | - | - | NI | - | 0.5±0.10 | 1.15±0.33 | 1.47±0.86 | 2.16±0.38 | - | - | | - | - | - |
| NI #25^g^ | - | 2070 | - | - | NI | - | 0.20±0.04 | 0.43±0.11 | 0.60±0.35 | 0.95±0.15 | - | - | | - | - | - |
| Longifolenealdehyde | 19890-84-7 | 2081 | - | MS | O | - | 0.03±.01 | 0.05±0.00 | 0.08±0.01 | 0.08±0.00 | - | - | | - | - | - |
| Beyerene | 3564-54-3 | 2108 | - | MS | T | - | 0.03±0.00 | 0.09±0.02 | 0.07±0.01 | 0.07±0.01 | - | - | | - | - | - |
|  |  | RIcal^b^ | RIref^c^ | Identification^d^ | Class^e^ | *C. sativus* ^f^ | | | | | *F. culmorum* ^f^ | | | | | |
| Compounds^a^ | CAS |  |  |  |  | 2 days | 7 days | 14 days | 21 days | 28 days | 2 days | 7 days | | 14 days | 21 days | 27 days |
| NI #26^g^ | - | 2133 | - | - | NI | - | 0.09±0.04 | 0.29±0.07 | 0.39±0.13 | 1.10±0.27 | - | - | | - | - | - |
| NI #27 ^g^ | - | 2141 | - | MS | NI | - | 0.10±0.03 | 0.26±0.06 | 0.36±0.21 | 0.59±0.10 | - | - | | - | - | - |
| Isopimaradiene | 1686-66-4 | 2144 | - | MS | T | - | 0.03±0.00 | 0.05±0.01 | < LOQ | < LOQ | - | - | | - | - | - |
| Isokaurene | 5947-50-2 | 2159 | - | MS | T | - | 0.27±0.05 | 0.45±0.10 | 0.38±0.05 | 0.28±0.02 | - | - | | - | - | - |
| Chemical classes  Alcohols  Organic esters  Aromatic hydrocarbons  Ketones  Alkanes  Alkenes  Furanic compounds  Suphur compounds  Terpenoids  Others  Non Identified |  |  |  |  |  | 87.30±53.18  -  -  -  -  -  -  -  12.70±10.64  -  - | 0.10±0.04  0.01±0.00  0.03±0.02  0.02±0.01  -  -  -  -  94.98±1.20  0.03±0.01  4.83±0.02 | 0.08±0.01  0.07±0.01  -  -  0.06±0.02  -  -  -  94.73±5.41  0.05±0.00  5.01±0.07 | 0.09±0.01  0.15±0.02  -  -  -  -  -  -  93.96±12.39  0.08±0.01  5.72±0.13 | -  0.32±0.03  -  -  -  -  -  -  90.36±1.76  0.08±0.00  9.24±0.07 | 35.86±10.6  -  -  1.07±0.00  10.62±1.10  -  -  -  13.68±0.61  38.77±3.91  - | 53.32±4.42  -  8.62±2.84  7.64±1.02  30.42±22.63  -  -  -  -  -  - | | 17.60±6.65  16.25±5.15  17.13±2.83  36.86±27.58  8.17±3.36  -  -  -  -  -  - | 13.79±0.81  20.19±10.7  18.43±5.48  39.09±32.04  8.5±6.75  -  -  -  -  -  - | -  30.66±23.25  24.10±1.72  29.31±0.76  15.93±16.86  -  -  -  -  -  - |

^a^ Compounds listed from their order of elution in a VF-Wax polar capillary column (30 m × 0.25 mm × 0.25 µm)

^b^ Linear retention index calculated on a VF-Wax capillary column with a homologous series of n-alkanes (C7–C30).

^c^ Linear retention index in literature.

^d^ Identification proposal is indicated by the following: MS, identification by comparing EI mass spectrum with Wiley275, pal600k and NBS75K mass spectral database; RI, identification by retention indexes with literature data; STD, comparison with the retention times and mass spectra of available standards.

^e^ Chemical classes: A: alcohol, E: organic ester, H: aromatic hydrocarbon, K: ketone, Aa: alkane, Ae: alkene, F: furanic compound, S: sulphur compound, T: terpenoid, O: other, NI: non identified

^f^ Mean percentage of VOCs emitted by 5 fungal cultures, for an initial fungal inoculum of 2,000 conidia.

^g^ Only the five major non-identified molecules (NI) of each treatment (> LOQ) were listed in the table.

< LOQ means that the mean quantity of the compound is below the limit of quantification (signal /noise ratio < 3 at the same retention time).
